# Field epidemiology training programmes in the Asia-Pacific: what is best practice for supervision?

**DOI:** 10.5365/wpsar.2019.10.1.007

**Published:** 2019-12-11

**Authors:** Owen Forbes, Stephanie Davis, Amalie Dyda, Alexander Rosewell, Stephanie Williams, Martyn Kirk, Maria Concepcion Roces, Maria Consorcia Lim-Quizon, Kerri Viney

## Abstract

**Introduction:**

Field epidemiology training programmes (FETPs) emphasize competency-based training and learning by doing. Supervision of FETP trainees is critical for programmes to achieve learning outcomes. We sought to address a knowledge gap regarding what constitutes effective FETP supervision.

**Methods:**

We investigated FETP supervision using a mixed-methods approach. Quantitative data were collected through a survey of FETP directors. Qualitative data included written feedback from the survey and a focus group discussion (FGD) conducted with FETP supervisors at the 8th South-East Asia and Western Pacific Bi-regional TEPHINET Conference. FGD questions focused on effective supervisory qualities and activities and challenges to effective supervision. We calculated descriptive statistics for quantitative data and analysed qualitative data using a deductive content analysis approach.

**Results:**

Eleven FETP directors responded to the survey and 23 participated in the FGD. Overall, supervision was seen as very important for trainee outcomes. Participants identified the different roles of academic and field supervisors but emphasized the importance of an enabling and supporting attitude towards trainees. Soft skills and interpersonal abilities were among the most important qualities identified for effective supervision. Key challenges identified included a lack of consistency in supervisors’ technical knowledge and the difficulty of finding candidate supervisors with sufficient interest, availability and motivation for supervision.

**Discussion:**

Several practical recommendations arose from this study for supervision in FETPs, including recruiting and training supervisors with a more holistic range of skills. Our findings also provide key points for current FETP supervisors to consider to improve their own practice.

Field epidemiology is defined as the “the practice of epidemiology in real time and real place, which in turn involves both science and art.” (*1*) Field epidemiology is a key component of the response to some of the world’s major public health problems; it has been vital to detecting and controlling such large-scale outbreaks as the 2014 outbreak of Ebola virus disease in West Africa, the 2009 H1N1 influenza pandemic and the 2003 multicountry outbreak of severe acute respiratory (SARS). Adequate training of field epidemiologists is an indicator of country capacity in implementing the International Health Regulations (2005). (*2*)

Field epidemiologists are frequently trained in dedicated on-the-job training programmes called field epidemiology training programmes (FETPs). (*3*) FETPs aim to build the capacity of public health systems in the countries where they are implemented. They do this by recruiting health-care workers, scientists and others and building their competence in field epidemiology through on-the-job mentorship, supervision and training. Several practices distinguish these programmes from academic education in public health. FETPs use a service-based approach (where trainees support the host organization’s priorities), implementing competency-based training  under the supervision of qualified mentors/supervisors and strengthening systems capacity using a learning-by-doing approach. (*3*) Following the establishment of the United States Epidemic Intelligence Service as one of the first formal FETPs in 1951, other FETPs have been established worldwide. (*4*) The Training Programs in Epidemiology and Public Health Interventions Network (TEPHINET) reports that there are now 71 FETPs  operating in more than 100 countries globally. (*5*)

Supervision of trainees in the field is a core component of FETPs and one that is thought to facilitate learning and the application of that learning to promote and protect public health. Supervisory structures vary according to the model of the FETP, the organization delivering the programme and the context. The FETP handbook identifies supervision as the responsibility of both technical leaders (typically resident advisers) and field supervisors. (*3*) Within this handbook, supervision is described as involving consultation on epidemiology methods, monitoring and evaluating trainee activities and mentoring and troubleshooting trainee projects. While terminology and models of supervision differ among programmes, it is typical for trainees to have a workplace or field supervisor who is based in their host organization in addition to an academic or programme supervisor who is a subject matter specialist affiliated with the FETP. For example, in many United States Centers for Disease Control and Prevention (CDC) programmes that provide support for FETPs, resident advisers (CDC epidemiologists employed to provide technical support to FETPs) and other programme staff provide additional scientific support to trainees, complementing day-to-day field supervision by a senior colleague in the workplace. (*3*) (Note that throughout this paper we use the term supervisor for anyone in a designated supervisory role of the trainee, whether this be a workplace/field supervisor or academic/programme supervisor. We recognize that in many programmes the term mentor is more frequently used for people in this role. Trainees also have many other names, but we have used trainees throughout.)

Despite the central role of supervision in FETPs, there is little published evidence on best practice in FETP supervision. Existing FETP guidelines largely focus on the logistical aspects of supervision with limited consideration of the broader qualities and activities that are important for effective supervision. (*3*, *6*) Programme experiences from the Asia-Pacific and other regions highlight issues related to FETP supervision such as a lack of adequate epidemiology knowledge among workplace supervisors, (*6*, *7*) and the interest in professional training in supervisory techniques. (*7*) A more comprehensive body of peer-reviewed evidence exists for other professions where supervision is a key component, such as health-care worker training that takes place in clinical settings. (*8*, *9*) Some of this literature may also be applicable to the FETPs, including the importance of integrating theoretical knowledge with practical experience (*10*) and recognizing the value of a holistic supervisory role, including interpersonal skills, nurturing and guiding alongside teaching specific skills and content knowledge. (*8*) However, none of the literature captures the particular needs and expectations of FETP supervisors.

Therefore, we undertook a mixed-methods study to determine the components and characteristics of effective supervision in the FETP context from the perspective of experienced FETP staff in the Asia-Pacific region. Our aim was to provide information on best practice in FETP supervision to further strengthen FETPs and the response to public health problems and threats.

## Methods

### Study design

This study employed a mixed-methods design, combining focus group discussions (FGDs) and a cross-sectional survey.

### Study population and data collection

FGD were held at a workshop for FETP supervisors titled “How to improve field epidemiology training in the Asia-Pacific” at the 8th South-East Asia and Western Pacific Bi-regional TEPHINET Conference, in Siem Reap, Cambodia from 28 November to 2 December 2016. The workshop was advertised as “of interest to FETP staff and supervisors” via materials sent to all attendees before the conference. Two experienced facilitators from Australia presented a summary of the literature on FETP supervision, including knowledge gaps; then, the facilitators guided FGD on the key themes outlined in **Box ****1**. These discussions were documented in detailed notes.

**Table 1:** FETP directors’ ratings of effectiveness of different supervisory activities (*n* = 11)

Supervisory activities	Rated “effective” or “very effective” *n*(%)	Rated “neither effective nor ineffective” *n*(%)	Rated “ineffective” or “very ineffective” *n*(%)
**Ensuring the trainee has adequate exposure to outbreaks and other public health events**	**11 (100%)**	**0 (0%)**	**0 (0%)**
**Teaching specific techniques and concepts, e.g. teaching how to write a questionnaire**	**10 (91%)**	**1 (9%)**	**0 (0%)**
**Ensuring the trainee has appropriate level of responsibility**	**10 (91%)**	**1 (9%)**	**0 (0%)**
**Ensuring the trainee has an adequate amount of time to complete work**	**10 (91%)**	**1 (9%)**	**0 (0%)**
**Regular one-on-one meetings with formal feedback**	**10 (91%)**	**0 (0%)**	**1 (9%)**
**Informal on-the-job feedback**	**9 (82%)**	**2 (18%)**	**0 (0%)**
**Observation by the trainee of the supervisor conducting outbreak investigations**	**9 (82%)**	**1 (9%)**	**1 (9%)**
**Giving advice on professional development**	**9 (82%)**	**2 (18%)**	**0 (0%)**
**Observation by the supervisor of the trainee conducting outbreak investigations**	**9 (82%)**	**2 (18%)**	**0 (0%)**
**Feedback on written work**	**8 (73%)**	**3 (27%)**	**0 (0%)**

**Table:** Box 1. Key themes addressed in the workshop and survey

What are the roles of a Field Epidemiology Training Programme (FETP) supervisor?
What makes a good FETP supervisor?
What are the challenges to supervision?

To obtain quantitative insights on different aspects of the key topic areas, FETP directors attending the same TEPHINET conference were invited to provide their views in a survey on various aspects of FETP supervision. The survey included 16 open- and closed-ended questions about FETP supervision including the differing roles of programme and field supervisors, effective supervisory qualities and activities, and challenges to supervision. Survey questions were based on key themes and findings about supervision in the literature review and informed by reports from the investigators who are experienced FETP supervisors. (*8*, *9*, *11*)

### Data analysis

Qualitative data from the workshop were analysed using a deductive content analysis approach. (*12*) Quantitative survey data were analysed using descriptive statistics. We calculated numbers and proportions of positive responses for questions with binary responses; Likert scale questions were calculated using the numbers and proportions of positive, negative and neutral responses. Data from open-ended questions were coded according to the framework of themes developed from workshop data (**Box ****1**).

### Consent

Participants at the workshop signed consent forms to participate in the workshop and have the findings published. FETP directors provided implied consent by responding to the survey and were informed that the results of the survey would be published.

### Ethics statement

Ethics approval for this study was provided by the  Australian National University (protocol 2016/420) and the University of New South Wales Human Research Ethics Committees (protocol 15571).

## Results

Results are organized into themes addressing the key study questions, shown in **Box ****1**. Quantitative results from the survey are presented in **Tables 1**** to ****4**. Qualitative analyses of workshop discussions are available in **Box ****2**. The majority of survey and FGD questions did not distinguish between field and programme supervisors. Other than in **Table 2**, results describe a general perspective on good practice in supervision without reference to specific supervisory roles.

**Table 4:** FETP directors’ ratings of important challenges to supervision (*n* = 11)

Challenges to supervision	Rated “somewhat important” or “very important,” *n*(%)	Rated “neither important nor unimportant,” *n*(%)	Rated “not very important” or “not at all important,” *n*(%)
**Supervisor lacks interest in supervision**	**11 (100%)**	**0 (0%)**	**0 (0%)**
**Supervisor lacks skills and knowledge on how to give effective feedback**	**11 (100%)**	**0 (0%)**	**0 (0%)**
**Supervisor lacks technical skills and knowledge in public health**	**10 (91%)**	**1 (9%)**	**0 (0%)**
**Supervisor lacks time to devote to supervision**	**9 (82%)**	**2 (18%)**	**0 (0%)**
**Supervisor is an inappropriately senior person within the organization**	**8 (73%)**	**1 (9%)**	**2 (18%)**

**Table 2:** FETP directors’ ratings of key supervisory activities for field and programme supervisors

Supervisory activity	Rated positive for field supervisors, *n*(%), *n* = 11	Rated positive for programme supervisors, *n*(%), *n* = 10
**To teach the trainee interpersonal skills (i.e. teamwork)**	**11 (100%)**	**4 (40%)**
**To help the trainee negotiate organizational/logistical issues**	10 (100%)*	4 (44%)*
**To help the trainee develop professional networks**	10 (100%)*	7 (78)*
**To provide opportunities for the trainee to develop technical skills (i.e. data analysis) but not directly teach these**	**10 (91%)**	**8 (80%)**
**To provide opportunities for the trainee to develop interpersonal skills (such as teamwork) but not directly teach these**	**10 (91%)**	**6 (60%)**
**To monitor the trainee’s progress**	**10 (91%)**	5 (56%)*
**To provide feedback to the trainee on their progress**	**10 (91%)**	**6 (60%)**
**To ensure the quality of outbreak investigations undertaken by the trainee**	**10 (91%)**	**7 (70%)**
**To motivate the trainee**	9 (90%)*	5 (56%)*
**To identify projects for the trainee**	9 (90%)*	6 (67%)*
**To inspire the trainee**	8 (80%)*	**5 (50%)**
**To provide emotional support to the trainee**	8 (80%)*	**4 (40%)**
**To help the trainee recognize their strengths and weaknesses**	8 (80%)*	7 (78%)*
**To teach the trainee technical skills (i.e. data analysis)**	**8 (73%)**	**8 (80%)**
**To teach the trainee management skills (i.e. managing staff)**	**8 (73%)**	**5 (50%)**
**To ensure the quality of other public health work undertaken by the trainee**	**8 (73%)**	**6 (60%)**
**To provide opportunities for the trainee to develop management skills (i.e. managing staff) but not directly teach these**	**7 (64%)**	**5 (50%)**
**To help the trainee develop a career plan**	**6 (55%)**	**5 (50%)**

* = 1 response missing for this item

**Table:** Box 2. Key findings arising from the discussion on FETP supervision at the 8th South-East Asia and Western Pacific Bi-regional TEPHINET Conference workshop, 2016

What are the roles of a field epidemiology training programme (FETP) supervisor?
Helping trainees meet programme requirements, including:
Logistical support
directing trainees to appropriate projects
arranging access to data and appropriate resources, including people
Providing feedback
helping trainees improve their performance and ultimately meet their programme requirements
Teaching and training
supporting the trainee to learn by doing rather than teaching didactically
Different roles in different settings
instructive, technical support in the classroom and more pragmatic, personal support in the field
Helping the trainee to develop a professional network
supporting trainees to build networks within public health and epidemiology communities
Holistic support of the trainee
assisting trainees to develop skills and face challenges in both work and life and overall supporting an enriching trainee experience

What makes a good FETP supervisor?
Skills and qualities
interpersonal skills, mentoring and leadership skills, patience, commitment and motivation in supervision, empathy for trainees, role modelling and guidance
**Availability** *easily available and approachable, with plenty of time made for trainees*
Professional background
having a health-related profession and training to confer technical knowledge and credibility
Understanding of FETP requirements
having a sound understanding of programme requirements and expectations of trainees
Different styles for different settings
being able to adapt supervisory style according to trainee needs and contextual demands

What are the challenges to supervision?
Time and availability
supervisors lacking time and availability is a major barrier to positive trainee outcomes
Differing expectations from different supervisors
when supervisors disagree and particularly when they give diametrically opposite feedback, this can be very challenging for both trainees and supervisors
Cultural and language issues
for supervisors and trainees working in cross-cultural environments, there can be challenges around expectations of interactions and how feedback is given and received
Organizational barriers and issues
issues can arise when trainees are in employed positions and have a workplace supervisor who may not understand FETP projects and deliverables and may also want the trainee to do other work
Doing rather than enabling
trying to didactically teach trainees the right way to do something can be a frustrating and time-consuming process, rather than supporting them to guide their own learning needs

### Participation in the survey and workshop

Twenty-three participants attended the workshop. Of the 19 total member countries represented in the Western Pacific and South-East Asia regions of TEPHINET, participants were associated with programmes in 13 countries and identified themselves as supervisors, mentors and resident advisers.

Directors from 11 of these TEPHINET programmes responded to the survey (response rate of 58%). Of these, 91% (*n* = 10) had a programme duration of 13–24 months. The majority of programmes (73%, *n* = 8) had been established for more than 10 years, 18% (*n* = 2) for 6–10 years and 9% (*n* = 1) for 4–5 years.

### What are the roles of an FETP supervisor?

#### Survey

Overall, FETP directors appeared to have a high degree of recognition of the value of supervisors for effective FETP training. The majority of FETP directors (55%) perceived the role of the supervisor as being “very effective” in facilitating the development of competent field epidemiologists; the remainder rated the supervisor’s role as “effective.”

A wide range of activities were considered to be part of the supervisory role (**Tables 1**** and ****2**). The activities rated as most important for effective supervision were those that emphasized an interpersonal connection between the supervisor and trainee to learn practical skills, including logistical arrangements to support these activities. Comparing the roles of field and programme supervisors, the field supervisor was seen as having a more holistic role with more than 50% of participants rating each of the 18 activities outlined in **Table 2** as part of the field supervisor’s role. In contrast, many participants perceived the programme supervisor to have a more defined role that focused on transfer of technical skills and knowledge. One survey participant indicated that “daily discussions on outbreak investigation and feedback to the trainee” were a particularly effective supervisory activity, reflecting the strong emphasis on the value of interpersonal contact and soft skills developed through regular contact with field supervisors (**Table 2**).

#### Workshop

A key theme from the workshop was that the primary role of the supervisor was not to teach didactically, but instead to facilitate the trainee’s learning and to guide the trainee to ask the right questions. This was seen as fitting under the overall ethos of FETPs, facilitating learning by doing, and was exemplified by one participant’s analogy of the supervisor as a midwife: the supervisor coaches and provides encouragement and expertise, but in the end, it is the trainee who has to do the work.

Another key theme from the workshop was the  different supervisory roles played by programme and field supervisors. Several participants highlighted that, in the classroom, supervisors should act mainly as instructors in specific technical areas. In the field, however, their role was to supervise the work of the trainee in outbreak investigations and to provide technical support, if needed. It was suggested that these differences could limit the capacity for programme supervisors to provide support on more holistic or interpersonal matters, while field supervisors held greater responsibility for supporting development of a different set of skills such as leadership and communication.

### What makes a good FETP supervisor?

#### Survey

FETP directors perceived the supervisor’s level of public health knowledge as the most critical quality for effective supervision (**Table 3**). Similarly, their technical skills were also seen as highly important along with a range of more holistic qualities including enthusiasm, interpersonal skills, approachability and availability.

**Table 3:** FETP directors’ ratings of the importance of selected supervisor qualities and skills (*n* = 11)

Supervisor qualities and skills	Rated “somewhat important” or “very important,” *n*(%)	Rated “neither important nor unimportant,” *n*(%)	Rated “not very important” or “not at all important,” *n*(%)
**Public health knowledge**	**11 (100%)**	**0 (0%)**	**0 (0%)**
**Technical skills**	**10 (91%)**	**1 (9%)**	**0 (0%)**
**Enthusiasm for public health**	**10 (91%)**	**1 (9%)**	**0 (0%)**
**Interpersonal skills**	**10 (91%)**	**1 (9%)**	**0 (0%)**
**Approachability**	**10 (91%)**	**1 (9%)**	**0 (0%)**
**Availability**	**10 (91%)**	**1 (9%)**	**0 (0%)**
**Enthusiasm for teaching**	**9 (82%)**	**2 (18%)**	**0 (0%)**
**Empathy**	**8 (73%)**	**3 (27%)**	**0 (0%)**
**Seniority**	**5 (45%)**	**6 (55%)**	**0 (0%)**

#### Workshop

The workshop discussion highlighted that good interpersonal skills, particularly mentoring and leadership skills, as well as high levels of patience were particularly important for effective supervision. Good supervisors were seen as being committed and having passion and motivation to supervise. Other participants viewed the supervisory relationship as a type of parental role in which the supervisor cared about and guided the trainee in a range of areas. Workshop participants identified a supervisor’s level of availability and approachability as critical characteristics.

In addition to their personal and interpersonal traits, the training background of the supervisor – specifically having a health-related profession – was seen as important. In addition to presumably conferring technical knowledge, having a health-related background was seen to confer credibility to the supervisor. It was also seen as critical that supervisors had a sound understanding of programme requirements so they knew what the trainee was expected to achieve.

### What are the challenges to supervision?

#### Survey

A lack of interest in supervision and insufficient skills to provide effective feedback were perceived to be the greatest challenges to effective supervision (**Table 4**). A survey participant noted that “not all can effectively teach, even though they may be able/competent,” suggesting that aptitude for and interest in supervision are important along with a supervisor’s level of knowledge and experience.

A lack of technical skills and relevant knowledge was seen as the next most critical barrier to good supervision (**Table 4**). A supervisor’s lack of time was also noted as a challenge, and comments from survey participants also stated that supervisors often lacked time to support trainees. Other comments identified the remoteness of programme supervisors and their lack of time spent face to face with trainees as a barrier to teaching technical competencies. The supervisor’s degree of seniority was seen as less important, with only 27% (***n*** = 3) identifying this as a “very important” challenge for effective supervision (**Table 4**).

#### Workshop

It was deemed important to engage committed and motivated supervisors. However, identifying and finding these people was perceived to be challenging. This was linked to the issues of time and availability, as people who would make good supervisors may lack the time required for hands-on supervision.

Differing expectations between supervisors across contexts was a commonly reported issue. It was acknowledged that having multiple supervisors is sometimes necessary, but when supervisors disagree, it can be challenging for both trainees and supervisors. Different expectations of the trainees can also be an issue when trainees are in employed positions and report to a superior who is not FETP trained and who may not fully understand the trainees’ projects and deliverables.

Participants identified various barriers and challenges to giving effective feedback to trainees. These included different expectations between supervisors and trainees, cultural and language barriers, availability of trainees and supervisors for meetings due to physical locations and time available from other responsibilities. Professional reflective learning, clear expectations about required programme standards as well as operating within a trusting supervisory relationship were seen as important in trying to manage these barriers and challenges.

## Discussion

Workshop and survey results indicated that FETP directors and supervisors had a high level of confidence in the value and effectiveness of FETP supervisors. Participants identified several key components to effective supervision, including interpersonal and communication abilities, relevant training background and technical skills and time and availability for frequent in-person contact with trainees. We found several areas to improve the structure and practice of supervision in FETPs.

Existing FETP guidelines often focus on the specific logistical and didactic responsibilities of supervisors and emphasize the need for strong technical skills. (*3*, *13*) Our results highlighted the importance of a holistic role for the supervisor, which includes mentoring the trainee in interpersonal and communication skills, alongside technical competencies and knowledge. These findings are consistent with the literature from other field-based training such as clinical settings where priority is placed on supervisor reassurance, role modelling, empathy and interpersonal skills (in addition to technical skills); (*8*, *14*) the literature on academic supervision also emphasizes the importance of soft skills such as encouragement, empathy and supportiveness. (*15*)

The survey results highlighted important differences in the perceived roles of programme and field supervisors. One person can sometimes perform both roles and/or the roles may overlap; however, in our findings, the role of programme supervisor was seen to be more specific to teaching technical skills and knowledge. On the other hand, field supervisors were expected to provide practical, motivational and emotional support in addition to supporting learning. The many supervisory priorities reflect and are likely guided by existing FETP guidelines that outline the different roles of supervisor. (*3*, *13*) However, the most critical activity for both types of supervisor was viewed as supporting the trainees to develop their own knowledge and abilities rather than trying to make them learn the right way. While this reflects a key ethos of FETPs, i.e. learning by doing, it also reflects literature on best practice supervision in other areas. Academic  supervision literature provides similar guidance, suggesting the value of letting supervision be driven by the trainee’s needs (*15*) and striking a balance between direction and self-direction based on a trainee’s level of familiarity and expertise. (*16*) The literature on clinical supervision provides a similar view, suggesting that as trainees develop expertise they may benefit from independently directing their own learning and contribute to their own professional growth. (*9*) Overall, our study findings suggest that a key contribution of the supervisor is to enable trainees to identify and pursue areas for their own development, giving them opportunities to direct their own learning and to apply theoretical knowledge in practical scenarios (*10*) rather than taking a purely instructive approach.

Our study participants identified a variety of challenges to effective field epidemiology supervision, including: a lack of commitment and interest in being a supervisor; and ineffective communication skills, including the inability to provide constructive feedback. Another key challenge identified was that workplace supervisors could lack sufficient technical skills and knowledge, limiting their ability to provide adequate technical supervision. These experiences echo those reported from other programmes where poor training outcomes were reported from field supervisors who lacked any relevant background in public health or epidemiology. (*6*, *7*)

Our findings on both the challenges of supervision as well as the role and quality of good supervision have practical implications. They suggest that the ideal supervisor has a relevant background; well developed technical skills; good programme knowledge; is interested, warm, motivated and committed to FETP supervision; and has sufficient time to dedicate to these tasks. While this ideal may not be frequently realized, it is worthwhile for programmes to consider some of these qualities in supervisor recruitment. Programmes should also consider these traits when conducting orientation and training of supervisors; such training should cover programme requirements and operation but also help supervisors improve technical competencies. (*7*, *17*) As suggested in the clinical supervision literature, (*9*) supervisor training should also include opportunities to assess and train new supervisors in areas of effective communication, giving feedback, building trusting relationships and empathetic mentoring with trainees. The similarities of good supervisors alongside the challenges of supervision were remarkably similar between the programmes represented in this study, suggesting the value of inter-FETP collaboration to develop role descriptions and training for FETP supervisors. These could then be adapted to the local context and be included in programme curricula and guidelines for each country to enable supervisors to have greater understanding and expectations of their role.

A limitation to this study was that our sample was small and purposive. Given the specific nature of our research question, this was deemed the most feasible and appropriate study design. We did not collect data from the participants to assess the extent to which each participant was involved with direct supervision. However, individuals in the director role would typically have frequent contact with programme supervisors and substantial exposure to supervision practices. Another limitation was that we only considered the views of  supervisors rather than trainees and that we relied on self-reported subjective data. While this method is commonly used to assess the effectiveness of supervision across a range of settings, more objective evidence could be obtained by targeting evaluation at the level of subsequent trainee behaviour in the workplace or public health outcomes resulting from the work of FETP graduates. (*18*) Studies of supervision in other contexts have assessed efficacy using measures such as trainees’ publication rates and job attainment in relevant specialist fields, which could be explored in developing indicators of supervisor performance in FETPs. (*19*) Given the study’s sample from Western Pacific and South-East Asian FETPs, this may limit the generalizability of our findings to other regions, though comparison with other findings from African and Asia-Pacific FETPs indicates similar experiences and challenges with regards to supervision. (*6*, *7*)

To conclude, supervision is a core component of FETPs, and this study has identified some of the key elements and challenges of effective supervision in these programmes. Our findings provide the basis for practical recommendations for FETP supervisory recruitment and training. The findings also provide material for FETP supervisors to consider in their own reflective practice, including practising empathy towards their trainee’s overall professional and personal development and being responsive to both their practical and emotional needs. To better understand what and how effective supervision occurs, it would be worthwhile conducting further research in this area, particularly incorporating trainee viewpoints as well as evaluating supervision via FETP trainee outcomes.
